# Electrophysiological Correlates of Emotional Content and Volume Level in Spoken Word Processing

**DOI:** 10.3389/fnhum.2016.00326

**Published:** 2016-07-04

**Authors:** Annika Grass, Mareike Bayer, Annekathrin Schacht

**Affiliations:** ^1^Courant Research Centre Text Structures, University of GöttingenGöttingen, Germany; ^2^Leibniz-ScienceCampus Primate CognitionGöttingen, Germany

**Keywords:** emotional content, spoken word processing, volume level, ERPs, EPN, N1, P2

## Abstract

For visual stimuli of emotional content as pictures and written words, stimulus size has been shown to increase emotion effects in the early posterior negativity (EPN), a component of event-related potentials (ERPs) indexing attention allocation during visual sensory encoding. In the present study, we addressed the question whether this enhanced relevance of larger (visual) stimuli might generalize to the auditory domain and whether auditory emotion effects are modulated by volume. Therefore, subjects were listening to spoken words with emotional or neutral content, played at two different volume levels, while ERPs were recorded. Negative emotional content led to an increased frontal positivity and parieto-occipital negativity—a scalp distribution similar to the EPN—between ~370 and 530 ms. Importantly, this emotion-related ERP component was not modulated by differences in volume level, which impacted early auditory processing, as reflected in increased amplitudes of the N1 (80–130 ms) and P2 (130–265 ms) components as hypothesized. However, contrary to effects of stimulus size in the visual domain, volume level did not influence later ERP components. These findings indicate modality-specific and functionally independent processing triggered by emotional content of spoken words and volume level.

## Introduction

From an evolutionary perspective, the rapid detection of threats or life-sustaining opportunities is important for survival and fast adaptation and explains the outstanding importance of emotional stimuli for humans. The organization of the emotional response systems has been suggested to be founded on two basic motivation systems, an appetitive and a defensive system (Lang et al., 1997; Lang and Bradley, 2010). Reacting fast to a positive stimulus, for instance, might maximize the probability of attaining a rewarding state, whereas emotionally negative stimuli are best dealt with by initiating a rapid response that probably aids survival. Therefore, it seems conceivable that the high importance of emotional content shapes perceptual processing and finally results in appropriate reactions. Next to somatic reactions, this modulation is evident on the behavioral level in better memory performance (Kissler et al., 2007, 2009; Bayer et al., 2011), faster response latencies (Keil et al., 2005; Schacht and Sommer, 2009a,b; Bayer et al., 2011), and higher accuracies (Schacht and Sommer, 2009b) for emotional compared to neutral stimuli. The preferential processing of emotional stimuli is also evident in event-related brain potentials (ERPs). An ERP component being modulated by emotional content of stimuli from different domains is the early posterior negativity (EPN). The EPN is a relative negative deflection at posterior electrodes, which becomes visible approximately 200–300 ms after stimulus onset. The EPN has been linked to a boost of visual encoding due to enhanced attention allocation to emotional stimuli in comparison to neutral stimuli (Schupp et al., 2007; Junghöfer et al., 2001). Modulations of EPN amplitudes were shown for pictures depicting emotional relevant scenes and objects (Schupp et al., 2004, 2007; Bayer and Schacht, 2014) as well as for facial expressions of emotion (Holmes et al., 2008; Rellecke et al., 2012; Recio et al., 2014). However, the EPN was not only shown to be elicited by pictorial stimuli but also by written emotional words (Kissler et al., 2007, 2009; Schacht and Sommer, 2009a,b; Scott et al., 2009; Palazova et al., 2011, 2013; Bayer et al., 2012a; Opitz and Degner, 2012; Citron et al., 2013).

Emotional valence also seems to interact with the perception of proximity: positive objects are being perceived as closer than negative and neutral ones (Valdés-Conroy et al., 2012); and the effect of proximity on reaction times was shown to be modulated by the valence of an approaching stimulus (de Haan et al., 2016). Codispoti and De Cesarei (2007) investigated physiological changes and subjective ratings of participants in response to emotional pictures of varying sizes, as an increase in object size seems to be the main characteristic of an approaching object. They found an interaction of stimulus size and emotional reactions: pictures of large size triggered stronger emotional reactions than smaller pictures, consisting of increased amplitudes of skin-conductance responses as well as more pronounced differences in subjective valence and arousal ratings between emotional and neutral pictures. A similar interaction of emotion and stimulus size was found for the EPN, which started earlier and was more pronounced for large than for small pictures (De Cesarei and Codispoti, 2006). The authors proposed that an increase in image size might lead to enhanced emotional reactions due to the more direct biological relevance of pictorial stimuli. One could argue that the size of the picture reflects the subjective proximity of a perceiver to a given object in reality and thereby influences its biological relevance. For example, an aggressor is more dangerous the closer it is, and governed by the higher motivational relevance, the response to this stimulus should be more pronounced.

Independent of emotional aspects, N1 and P1 amplitudes are comparably modulated by both objects in near space (Kasai et al., 2003; Valdés-Conroy et al., 2014) and by bigger images (Nakayama and Fujimoto, 2015; Pfabigan et al., 2015), indicating a close link between image size and proximity. Similarly, these early stages of perceptual processing were shown to be impacted by other stimulus features as brightness, contrast, and texture appearance (Johannes et al., 1995; Balas and Conlin, 2015; Schettino et al., 2016).

Bayer et al. (2012a) investigated whether the interaction of stimulus size and emotion effects generalizes to linguistic materials, namely to isolated words of emotional meaning. If the interaction of image size and emotion existing for pictures is resulting from the higher biological relevance due to its direct resemblances of the object they depict, a similar effect would be unlikely to occur for written words, since they are entirely arbitrary and symbolic. Interestingly, large stimulus size—more precisely font size of written words—led to augmented ERP effects of emotional content in the EPN time window, showing high similarity to effects reported for affective pictures (De Cesarei and Codispoti, 2006). The authors thus concluded that the mechanism responsible for interactions of emotional and stimulus-triggered attention might not be limited to biologically relevant stimuli, but might also be engaged in processing of symbolic stimuli. Thus, a more general type of stimulus relevance might play a causal role in the found interaction of size and emotional content. The authors suggested that the mechanisms of sensory facilitation were originally based on a biological, survival-relevant type of relevance, but might have generalized to written words, probably reflecting the high social relevance of language (Bayer et al., 2012a).

As a consequence, the question arises if and how this mechanism would apply to the spoken domain of language, which may play an even more important role in the everyday life of human beings. Given that an approaching object mainly changes in its physical size, the main characteristic of sounds in near vs. distant environment are differences in their loudness (volume level) (e.g., von Bekesy, 1949; Begault, 1991; for a review on auditory distance perception see Zahorik, 1996). Similar to stimulus size in the visual domain, volume level has been shown to modulate early cortical responses to auditory stimuli. An increase in volume level increases the N1/P2 peak-to-peak amplitude (Rapin et al., 1966; Beagley and Knight, 1967; Picton et al., 1970; Adler and Adler, 1991; Thaerig et al., 2008). However, to the best of our knowledge, it remains unclear whether there exist later effects of volume level on auditory-evoked potentials and if volume level might also interact with emotion effects, as it has been shown for the stimulus size of emotional pictures and written words. A candidate component for a possible interaction of volume level and emotional content would be an auditory EPN, which was proposed to be an equivalent to the visual EPN in the auditory domain (Mittermeier et al., 2011; Jaspers-Fayer et al., 2012; Grass et al., 2016). Next to the semantic content of a sentence or word, spoken utterances comprise a second communication channel, namely prosody. The tone and rhythm of a speaker’s voice can convey emotion as well and might be more innate than the learned, artificial meaning of words. Using auditory stimuli of varying emotional prosody and content, two studies (Mittermeier et al., 2011; Jaspers-Fayer et al., 2012) demonstrated a negative ERP component occurring in emotional compared to non-emotional paradigms. A recent study investigating the effects of emotional content of spoken words (Grass et al., 2016) demonstrated that ERP differences between emotional and neutral spoken words were highly similar to the visual EPN component in terms of their scalp distributions. However, the latency of this effect in the auditory domain was prolonged by about 200 ms, due to the incremental nature of spoken word stimuli. Source localizations of the visual as well as the auditory emotion-related ERP effects revealed comparable neural generators in the superior parietal lobule (SPL) and inferior parietal lobule (IPL; Grass et al., 2016). These findings are in line with the assumption of Jaspers-Fayer et al. (2012) that the SPL is commonly involved in generating both the visual EPN and its auditory counterpart.

Next to the EPN-counterpart in response to auditory emotional stimuli, evidence also suggests the existence of an equivalent to the late positive complex (LPC), which has reliably been shown to reflect sustained elaborate processing of emotional stimuli in the visual modality. An auditory LPC was reported for spoken words with emotional connotation (Ofek et al., 2013; Hatzidaki et al., 2015) and emotionally uttered words and sentences (Costanzo et al., 2013; Paulmann et al., 2013). Although there is evidence for some similarities between emotion-related ERP effects in the visual and auditory modality, it is noteworthy that these effects show pronounced differences in their temporal dynamics. Furthermore, strong differences in terms of the latency of emotion-related effects can also be found *within* the auditory modality: whereas emotional prosody conveys salience almost immediately and can thus modulate quite early components, for example the P2 (Paulmann and Kotz, 2008; Agrawal et al., 2012; Pinheiro et al., 2013; Schirmer et al., 2013), full semantic information of spoken words, including their emotional content, incrementally develops over time (Bradley and Lang, 2000). Therefore, the time course of effects for emotional meaning is rather difficult to compare to effects for emotional prosody, but also to effects of emotional meaning in the visual modality.

The aim of the present study was to investigate the interplay of volume level and emotion effects for the auditory domain of language. In the present study, we used the stimulus material of Bayer et al. (2012a)^1^. The words were spoken in neutral prosody by a trained female speaker and presented in two different volume levels. First, we expected effects of volume on the N1-/P2- complex. Whereas for the written domain of word processing effects of emotional content on early components as the P1 have been reported (Hofmann et al., 2009; Bayer et al., 2012b; Hinojosa et al., 2015), early emotion effects for the auditory modality were not expected due to the following reasons: first, to our knowledge there is no evidence for impacts of emotional content on early ERP components in the auditory domain, except for tone stimuli that were associated with emotion in conditioning paradigms (Broeckelmann et al., 2011, JoN) and effects of emotional prosody as reported before. Although explicit ratings as well as autonomous measures indicate high similarities between affective picture and affective sound processing in terms of perceived emotional arousal and valence (Bradley and Lang, 2000; Partala and Surakka, 2003), early ERP modulations to nonlinguistic affective sounds have not yet been reported. Thierry and Roberts (2007) implemented a combination of an oddball paradigm and a one-back matching task, in which neutral sounds were presented at two different volume levels (standard vs. deviants), additionally intermixed with unpleasant sounds presented at low volume level (deviants). Importantly, volume differences within the neutral stimuli impacted early ERP components (N1, P2) whereas effects of unpleasantness became evident only after about 300 ms. Second, the study of Bayer et al. (2012a)—using the same word stimuli and a highly similar paradigm as we employed in our study—did not show emotion effects at the P1 level in the visual domain. Third, in the present study, ERPs were measured to the words’ onsets. Thus, during initial processing stages—as reflected by the auditory N1-P2 complex—only very small amount of (semantic) information is available. This incremental nature of auditory processing of rather complex stimuli as words and sounds might also explain the absence of early effects in the study of Thierry and Roberts (2007).

Similar to previous reports, we expected an emotion-related ERP effect, consisting of an enhanced frontal positivity and posterior negativity between about 400 and 500 ms after stimulus onset (Grass et al., 2016). Assuming that this component is a functional equivalent to the visual EPN, volume level should modulate these emotion effects on the auditory EPN, similar to interactions reported for emotional pictures and written words. This modulation should be limited to sensory encoding, while no interactions at higher-order processing stages should occur (De Cesarei and Codispoti, 2006; Bayer et al., 2012a). In contrast, if the mechanism underlying the interplay of stimulus size and emotion is restricted to the visual modality, effects of emotional content and volume level in auditory word processing should be independent.

## Materials and Methods

### Participants

Data was collected from 31 female participants. Two data-sets had to be discarded due to excessive ERP-artifacts. The remaining participants had a mean age of 23.7 years (*SD* = 2.8 years), were all right-handed (Oldfield, 1971), native German speakers, and reported no neurological or psychiatric disorders. Participants reported normal hearing range, which was further ensured by a short, custom-made hearing test administered prior to the experiment in which subjects had to count single tones at different volume levels. Participation was reimbursed with course credit or 8 €/h.

### Stimuli

Stimuli consisted of 72 German nouns that were of positive, neutral, or negative valence (*n* = 24 each). The three emotion categories differed significantly in their valence ratings, *F*_(2,69)_ = 1362.67, *p* ≤ 0.001 (all rating values were drawn from the Berlin Affective Word List Reloaded, Võ et al., 2009); with lower ratings for negative compared to neutral, *F*_(1,46)_ = 725.7, *p* ≤ 0.001, and higher ratings for positive compared to both negative, *F*_(1,46)_ = 2446.8, *p* ≤ 0.001, and neutral words, *F*_(1,46)_ = 727.74, *p* ≤ 0.001 (for descriptive statistics see Table 1). Neutral words were significantly less arousing than positive and negative words, *F*s_(1,46)_ > 99.0, *p*s < 0.001 which did not differ from each other, *F*_(1,46)_ = 1.68, *p* = 0.202. Emotion categories were controlled with regard to imageability, word frequency, and the number of letters and syllables, all *F*s_(2,69)_ ≤ 1.

**Table 1:** **Descriptive statistics (Means and Standard Deviations) for linguistic and auditory parameters of word stimuli**.

Parameter	Positive	Neutral	Negative
**Valence**	2.1 (0.2)	0.3 (0.2)	−2.0 (0.3)
**Arousal**	3.3 (0.7)	1.9 (0.2)	3.5 (0.5)
**Imageability**	5.4 (0.8)	5.6 (0.4)	5.5 (0.6)
**Letters**	6.3 (1.9)	6.3 (1.2)	6.4 (2.1)
**Syllables**	2.0 (0.8)	2.0 (0.8)	2.1 (1.0)
**Frequency**	27.7 (32.0)	24.6 (29.2)	24.8 (20.5)
**Duration**	682.2 (123.6)	628.5 (99.3)	694.6 (149.3)
**F0 Range**	61.4 (24.2)	66.0 (14.1)	57.3 (14.9)
**Mean F0**	207.2 (8.8)	202.4 (6.8)	205.3 (8.5)
**Low volume level**	43.0 (1.9)	43.0 (1.2)	43.1 (1.7)
**High volume level**	55.8 (2.5)	56.0 (2.2)	56.1 (2.5)

*For all ratings, the ranges are: −3 to +3 (valence), 1–5 (arousal), 1–7 (imageability). Frequency is indicated as occurrence per 1 million words in the CELEX database. Note that these values refer to the written version of our word stimuli (Võ et al., 2009). Mean F0, F0 range, and duration were measured in Praat (Boersma and Weenik, 2009) and are indicated in Hertz and milliseconds, respectively. High and low volume level are given in decibel*.

Words were spoken by a trained female speaker in neutral prosody and were recorded on a PC workstation using Adobe Audition (Adobe Systems Software, Dublin, Ireland). In a first step, mean amplitudes for each word were normalized; the analysis of acoustic parameters was then performed using Praat software (Boersma and Weenik, 2009). Emotion categories did not differ in amplitude, mean F0 (fundamental frequency), F0 variability, F0 peak values, overall duration, and speed per syllable. Stimuli were presented in two sound volumes. Based on a pilot experiment, volume levels were adapted in such a way that stimuli were audible in the low volume condition and not too loud in the high volume condition, in order to prevent participants from startling. The mean amplitudes were 43.0 dB (*SD* = 1.6 dB) in the low volume condition and 56.1 dB (*SD* = 2.5 dB) in the high volume condition, measured by a professional sound level meter (SL-322; ATP Messtechnik GmbH), placed at the approximate position of participants’ heads. Maxima in volume level did not exceed 67 dB, and minima were above 35 dB; thus all words stimuli were presented within the normal range of human communication (e.g., Schwartz and Krantz, 2016). Importantly, volume levels did not differ as a function of emotion, *F*s < 1, while both volumes significantly differed between the two loudness conditions as intended, *F*_(1,138)_ = 1363.6, *p* < 0.001 (see Table 1).

### Procedure

The study was approved by the ethics committee of the Institute of Psychology at the University of Goettingen, Germany, and was conducted according to the Declaration of Helsinki. Before the beginning of the experiment, participants were acquainted with the experimental procedure and signed informed consent. After preparation of EEG recordings, participants were seated in a sound-attenuated chamber. Participants were facing a computer monitor at a distance of 100 cm while words were presented by two loudspeakers positioned at a distance of 133 cm from the participant’s ears. The experiment consisted of four experimental blocks; within each block, each word was presented once. Half of the words per block were randomly presented at high volume and the other half at low volume, in total resulting in two presentations of each word at each volume level. The assignment of words to volume levels changed after each block and the order of this assignment, i.e., whether the first presentation of a word was at high or low volume, was counterbalanced. Participants were instructed to listen attentively to the presented words. A one-back task was employed at random intervals (on average after every 9th trial) in order to ensure that participants were paying attention to the word stimuli during the experimental session. In these test trials, a word was displayed within a green frame on the screen. Participants had to indicate by button press whether this word was identical or different to the one they had heard before. By presenting the words in their written form, semantic processing of the words was ensured since the task could not be performed on the basis of perceptual matching. During the presentation of each spoken word, a fixation cross was presented on the screen, starting 1000 ms prior word onset and remaining visible for 2000 ms after word onset in order to avoid visual offset potentials. The inter-trial-interval (blank screen) had a length of 1000 ms, resulting in an overall trial length of 4000 ms.

### EEG Recordings and Preprocessing

The EEG was recorded with the Biosemi ActiveTwo (Biosemi, Amsterdam, Netherlands) system from 64 electrodes mounted in an electrode cap (Easy-Cap, Biosemi). Six additional electrodes were placed at the outer canthi and below both eyes in order to record the electrooculogram; two electrodes were placed at the mastoids. The common mode sense (CMS) active and the driven right leg (DRL) passive electrode were used as reference and ground electrodes, respectively^2^. Electrode offsets were kept below a threshold of ±20 mV. Signals were recorded at a sampling rate of 512 Hz and a bandwidth of 104 Hz. Offline, data was processed with the BrainVision Analyzer (Brain Products GmbH, Munich, Germany). The continuous EEG signal was re-referenced to average reference and segmented into epochs of 1200 ms, starting 200 ms prior to word onset. Blinks were corrected using the Surrogate Multiple Source Eye Correction as implemented in Besa (Brain Electric Source Analysis, MEGIS Software GmbH, Gräfeling, Germany); segments containing artifacts (5.4%) were rejected (voltage steps larger than 50 μV, 200 μV/200 ms intervals difference of values, amplitudes exceeding −150 μV/150 μV, and activity lower than 0.5 μV). The overall number of discarded trials per condition (volume level by emotion) ranged between 0 and 19 and did not differ between conditions, as indicated by a repeated-measures ANOVA, all *F*s < 0.1. Segments were referred to a 200 ms pre-stimulus baseline and averaged per subject and experimental condition.

### Data Analysis

**Figure 1:**
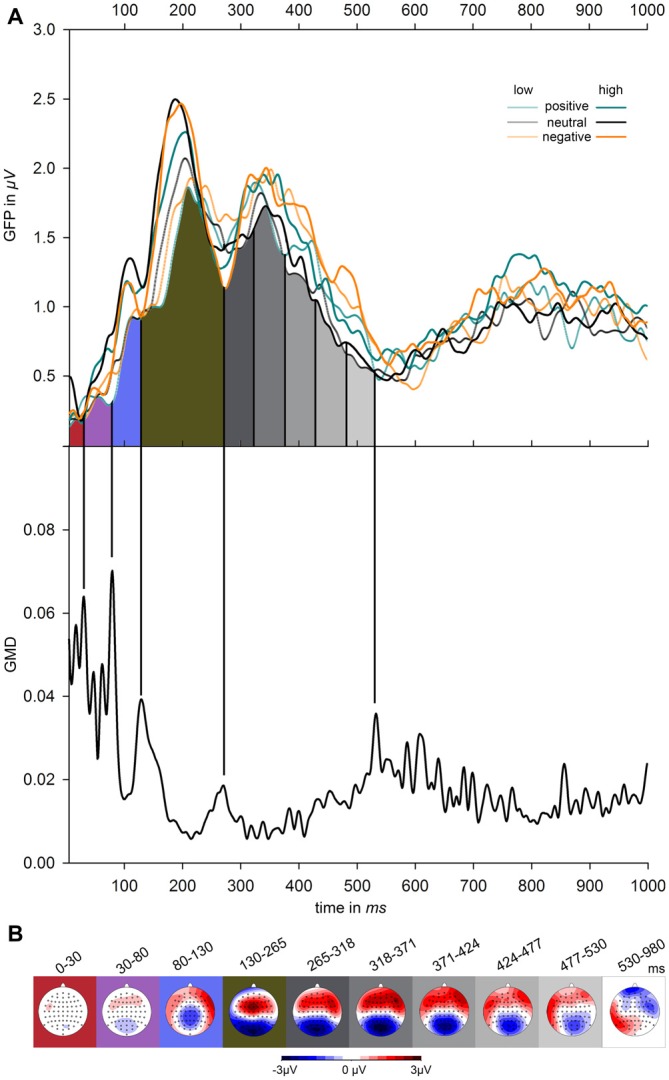
**Effects of emotional valence and volume level on electrophysiological parameters. (A)** The upper graph shows global field power (GFP) across all participants, contrasted for emotionally positive, negative, and neutral words presented at high and low volume level. The lower graph depicts global map dissimilarity (GMD) averaged across all subjects and experimental conditions. Vertical black lines mark the segment borders, which were defined according to the GMD peaks. Between the peaks at 265 ms and 530 ms, event-related potentials (ERPs) were divided into five equally long time windows and after the last clear peak at 530 ms in consecutive time windows of 50 ms between up to 980 ms. **(B)** Maps show the global scalp distribution averaged across all conditions during the time windows flanked by the borders depicted in **(A)**.

Segmentation of ERP amplitudes proceeded according to visual inspection of measures of global field power (GFP; Lehmann and Skrandies, 1980) and global map dissimilarity (GMD; Brandeis et al., 1992). Figure 1 depicts GFP contrasted for the factors emotion (positive, negative, neutral) and volume level (low, high), as well as GMD, which was calculated across the six experimental conditions. GFP reflects the overall ERP activity across the scalp at any given moment. GMD reflects the dissimilarity between scalp topographies of adjacent time points and demarcates the borders between periods of relatively stable topographies indicating continued processing within similar brain areas. These transition times were used as the limits of the time segments, for which mean ERP amplitudes were calculated. As becomes obvious from Figure 1, GMD peaks were clearly observable at the following time points 0, 30, 80, 130, 265, and 530 ms. In order to allow for more fine-grained analyses of ERPs during the interval of main interest, data was additionally sub-segmented between 265 and 530 ms into five time intervals of equal length (53 ms each). After the last clear segment border, consecutive time windows of 50 ms were analyzed between 530 and 980 ms. Amplitude differences were assessed by repeated-measures ANOVAs within these time borders, including the factors emotion (3–positive, negative, neutral) and volume level (2–high, low) and electrode (64). Degrees of freedom in ANOVAs were adjusted using Huynh–Feldt corrections. If indicated by significant electrode × emotion, electrode × volume level, or electrode × volume level × emotion interactions in these exploratory analyses, these effects were further tested in region of interests (ROIs) that were defined based on visual inspection of the ERP difference waves within the specific time windows. For *post hoc* comparisons, *p*-values were Bonferroni adjusted.

## Results

### Performance

Overall, participants performed highly accurate in the one-back task (percent correct = 99.6%, *SD* = 1.1).

### Effects of Volume Level

**Figure 2:**
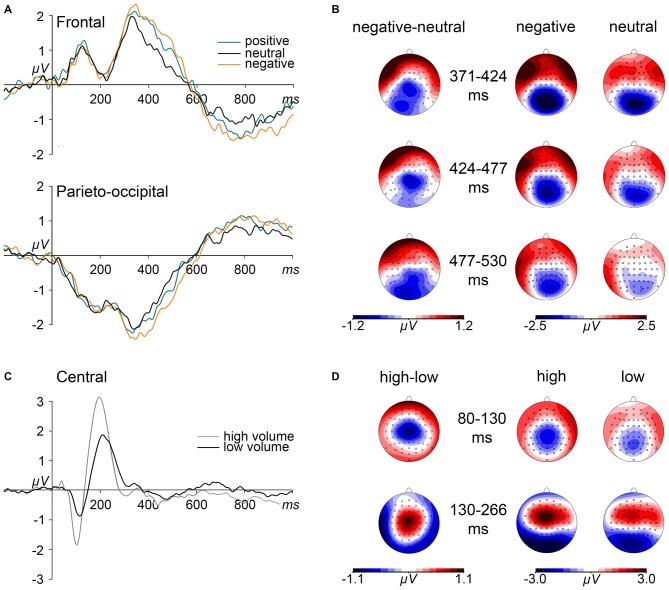
**Effects of emotional content and volume level on event-related potentials (ERPs). (A)** Grand mean ERP waveforms elicited by emotionally positive, negative, and neutral words are depicted from frontal and parieto-occipital region of interest (ROI) electrodes. **(B)** Depicted maps show the scalp distributions of the ERP differences between negative and neutral words within the time intervals of significant emotion effects as well as the distribution of ERPs, separated for negative and neutral words. **(C)** Grand mean ERP waveforms, contrasted for high and low volume level, are depicted for central ROI electrodes.** (D)** Maps depict the scalp distributions of ERP differences between high and low volume level words as well as the topographies of ERPs for both volume conditions within the indicated N1 and P2 time intervals.

Significant interaction effects of electrode × volume level were revealed in the two consecutive time windows between 80 and 130 ms, *F*_(63,1764)_ = 6.314, *p* < 0.001, $\eta_{p}^{2}$ = 0.184, and between 130 and 265 ms, *F*_(63,1764)_ = 8.948, *p* < 0.001, $\eta_{p}^{2}$ = 0.242. These interactions were driven by significant volume level effects in a central ROI (electrodes: C1, C2, Cz, CP1, CP2, CPz, FC1, FC2, FCz). As can be seen in Figures 2C,D, high volume words elicited more negative amplitudes as compared to low volume words between 80 and 130 ms, *F*_(1,28)_ = 45.456, *p* < 0.001, $\eta_{p}^{2}$ = 0.619, and more positive amplitudes between 130 and 265 ms, *F*_(1,28)_ = 45.453, *p* < 0.001, $\eta_{p}^{2}$ = 0.614.

### Effects of Emotion

The omnibus ANOVA revealed electrode × emotion interaction starting between 371–424 ms, *F*_(126,3528)_ = 2.570, *p* < 0.01, $\eta_{p}^{2}$ = 0.084, reflecting significant emotion effects in a frontal ROI (electrodes: AF3, AF4, AF7, AF8, AFz, FP1, FP2, FPz), *F*_(2,56)_ = 4.623, *p* < 0.05, $\eta_{p}^{2}$ = 0.142 and in a parieto-occipital ROI (electrodes: CPz, CP1, CP2, Pz, P1, P2, P3, P4, POz, PO3, PO4, O1, O2, Oz), *F*_(2,56)_ = 4.465, *p* < 0.05, $\eta_{p}^{2}$ = 0.138. As depicted in Figures 2A,B, spoken words of negative content elicited a stronger relative anterior positivity, *F*_(1,28)_ = 13.612, *p* < 0.01, $\eta_{p}^{2}$ = 0.327, and parieto-occipital negativity, *F*_(1,28)_ = 11.461, *p* < 0.01, $\eta_{p}^{2}$ = 0.290, than neutral words, while positive words only showed trends towards significance in the frontal ROI, *F*_(1,28)_ = 6.480, *p* = 0.051, $\eta_{p}^{2}$ = 0.188.

This emotion × electrode interaction sustained during the two consecutive time windows, i.e., between 424 and 477 ms, *F*_(126,3528)_ = 2.046, *p* < 0.05, $\eta_{p}^{2}$ = 0.068, and between 477 and 530 ms, *F*_(126,3528)_ = 1.993, *p* < 0.05, $\eta_{p}^{2}$ = 0.066. In the first interval, this interaction resulted from significant emotion effects in both the frontal, *F*_(2,56)_ = 3.680, *p* < 0.05, $\eta_{p}^{2}$ = 0.116, and the parieto-central ROI, *F*_(2,56)_ = 3.522, *p* < 0.05, $\eta_{p}^{2}$ = 0.112. These effects were driven by larger amplitudes to negative than neutral words in the frontal, *F*_(1,28)_ = 7.695, *p* < 0.05, $\eta_{p}^{2}$ = 0.216, and the parieto-occipital ROI, *F*_(1,28)_ = 8.111, *p* < 0.05, $\eta_{p}^{2}$ = 0.225. In the following interval (477–530 ms), an emotion effect again was discernible at the frontal as well as at parieto-occipital electrodes, *F*_(2,56)_ = 3.406, *p* < 0.05, $\eta_{p}^{2}$ = 0.108 and *F*_(2,56)_ = 5.761, *p* < 0.01, $\eta_{p}^{2}$ = 0.171, respectively. Negative words only showed a trend to elicit a frontal positivity, *F*_(1,28)_ = 6.497, *p* = 0.051, $\eta_{p}^{2}$ = 0.188 but significant effect at parieto-occipital electrodes, *F*_(1,28)_ = 10.975, *p* < 0.01, $\eta_{p}^{2}$ = 0.282.

Emotion × electrode interactions did not reach significance in any of the other time windows.

### Interaction Effects

Importantly, there was no three-way interaction of the factors emotion, volume level, and electrode in any of the time windows. Thus, in the present study, volume level did not modulate emotion effects.

## Discussion

The present study aimed at investigating the interplay of volume level and emotional content in spoken words. To this end, we presented words of positive, negative, and neutral content in two different volume levels while recording ERPs. As expected, volume level led to a modulation of early processing stages at level of the N1 (80–130 ms) and P2 (130–265 ms) component, confirming the well-known influence of intensity on N1/P2 peak-to-peak amplitude (Rapin et al., 1966; Beagley and Knight, 1967; Picton et al., 1970; Adler and Adler, 1991; Thaerig et al., 2008). The processing of words presented at higher volume level led to enhanced N1 and P2 amplitudes at central electrodes compared to low volume level words.

The processing of words of emotional relative to neutral content elicited emotion effects starting around 370 ms after stimulus onset. Negative emotional content led to an increased frontal positivity and a parieto-occipital negativity compared to neutral content between 371 and 530 ms. Only between 371 and 424 ms, a trend was discernible for positive words eliciting an enhanced anterior positivity compared to neutral words. These effects showed similarity to EPN topographies and might indeed resemble an auditory EPN as it was proposed to exist as an equivalent to the visual EPN (Mittermeier et al., 2011; Jaspers-Fayer et al., 2012; Grass et al., 2016). In the visual domain, the EPN component is assumed to reflect a boost in visual encoding due to enhanced attention allocation to emotional stimuli, mainly based on its temporal and topographical similarities to the so-called selection negativity (SN) triggered by voluntary attention allocation (Hillyard and Anllo-Vento, 1998; cf., Schupp et al., 2007).

However, compared to previous—particularly visual—EPN effects, the distribution of the emotion effect found in the present study expanded further towards central scalp areas. Thus, the effect of emotional content shows some similarity to the N400 component. The N400 is known as an indicator of semantic processing and was reported not only for visual, but also for auditory paradigms (e.g., Hahne and Friederici, 2002; Wambacq and Jerger, 2004; Diamond and Zhang, 2016). In the visual modality, it is modulated by the overall expectancy and congruity of (neutral) stimuli in semantic contexts (cf. Kutas and Federmeier, 2011), but also for words of negative content when embedded into sentences (e.g., Holt et al., 2009; Bayer et al., 2010). The one-back task employed in our study required a cross-modal comparison between visually presented catch stimuli and preceding spoken target words. Although the one-back trials incidentally occurred in about 10% of all trials, this paradigm might have spanned a very general semantic context, in which spoken words of negative content might have been less expected than words of neutral or positive emotional valence. However, we would like to point out that N400 effects recorded with comparable setups (mainly, average reference) usually occur with a more central maximum. Therefore, the ERP emotion effect found in our study might reflect a mixture of both components rather than a solely EPN-like or N400 component.

Interestingly, the factor volume level did not interact with the factor emotion. Although we found reliable effects of emotional content, showing anterior positivities and parietal to posterior negativities for emotional compared to neutral words, these effects were not modulated by the loudness of the presented words. This finding might indicate that the mechanism underlying interactions of emotional content and stimulus-triggered attention is acting across different stimulus domains in the visual modality (i.e., both for pictures and written words), but presumably not across different modalities. In the visual domain, comparable interaction effects of stimulus size and emotional content were found on the EPN component in response to emotional pictures and words (De Cesarei and Codispoti, 2006; Codispoti and De Cesarei, 2007; Bayer et al., 2012a). For the auditory modality, our study was not able to show such an interaction on a component, which might be interpreted as a functional equivalent to the visual EPN. Alternatively, one could assume that differences between volume levels might have been too small to elicit such differences since the volume bandwidth was limited to volume levels that were audible, but not too loud, in order to prevent participants from startling. However, participants’ reports indicated that volume levels were distinguishable, and the modulations of N1 and P2 amplitudes provide further proof that the volume level manipulation in itself was successful.

Importantly, emotion in the auditory modality is conveyed via two different channels: the content of an utterance and the tone of voice, hence the prosody, which both impact spoken language processing. Thus, it seems likely that emotional relevance in the spoken modality is not only conveyed by the content but also by the prosody of the utterance (Steinhauer et al., 1999; Wambacq and Jerger, 2004; Kotz and Paulmann, 2007). The words used in the present study were spoken in a neutral tone of voice in order to make them directly comparable to written words, which do not have this second communication channel. Presumably, what makes a heated argument even more emotional and relevant to us is not just a raising of the voice *per se*, but raising the voice with a meaningful prosody. Raising the voice *per se* would lead to a change in different acoustic parameters as rhythm, timbre, and pitch as contrasted with a distance-related increase in volume as has been proposed already by Gestaltpsychologists (e.g., Metzger, 1942). Thus, manipulating merely the loudness of the stimuli might not have heightened their relevance, and interactions of loudness and emotion might depend on corresponding changes of prosody.

In addition, the social context may play an important role in this paradigm. In our study, the words were played back via loud speakers without a speaker being visible. However, a recent study by Rohr and Abdel Rahman (2015) provided evidence that a more naturalistic context provided by the presence of a speaker’s face can strongly enhance emotion-related ERPs. Additionally, for visually presented words, previous studies demonstrated that context, especially self-reference and self-other discrimination, can enhance effects of emotion (Herbert et al., 2011a,b; Fields and Kuperberg, 2012). Due to these findings, it might be conceivable that interactions between volume level and emotional content might have occurred if the word’s relevance had been augmented by self-relevance or by multimodal presentation. Another possible explanation for the absence of interactions of emotion and loudness in the present study is the already mentioned incremental nature of auditory stimuli and the resulting poorer synchronization of the EEG signals across stimuli.

Importantly, the present results hint to a broader difference between visual and auditory language processing than the impact of volume on emotional processing. Notably, next to the absence of interactions between volume level and emotional content, there were no later main effects of volume level on ERPs. For visual stimuli of different domains, variation of stimulus size was shown to effect the EPN and the LPC (~400–600 ms; De Cesarei and Codispoti, 2006; Bayer et al., 2012a). In the present study, no effects of variation in volume level occurred after the P1-N2 complex. To our knowledge, most studies investigated the loudness dependence of the auditory evoked potential (LDAEP) only for the N1-P2 complex (Carrillo-de-la-Peña, 1999; Schadow et al., 2007; Park et al., 2010), but not for later components. Obviously, volume level impacts early perceptual processing (as reflected in N1 and P2), but might not be considered during high-order processing and stimulus evaluation. Probably, effects of stimulus size on later ERP components in the visual domain are resulting from the closer proximity perception. Presumably, in the auditory modality, effects of volume level on high-order processing components are missing since the proximity manipulation might be depending on more than this one factor and, for example, on visual input as well. Thus, volume level differences which are unaccompanied by “approaching” visual input might not be sufficient for a proximity manipulation and might therefore not impact high-order processing stages. It is conceivable that the proximity manipulation would have been enhanced by a speaker being present (at different distances); this question should be addressed in future research.

In conclusion, the present study replicated effects of emotional content on spoken word processing resembling an EPN-like component elicited by spoken words. However, neither this auditory EPN, nor any other investigated time window showed an interplay of sound level and emotional content, indicating that the mechanisms responsible for interactions of emotional content and stimulus-triggered attention in language processing might be limited to the visual modality. Overall, it should be mentioned that caution is advised when interpreting the absence of effects in the present study. Achieving an auditory proximity manipulation in an experimental setup where participants actually see the source of auditory stimuli might be another limitation of the present study. Future research is needed to prove the absence of the interaction effects with further evidence, in particular, for different types of emotional auditory stimuli.

## Author Contributions

AG analyzed the data and did the main writing of the manuscript, including figures, tables etc and revised it for important content. MB contributed to data analyses and together with AS developed the design and concept for this research. Both MB and AS were part of the drafting and revising process. MB and AG contributed equally to this work. All authors approved the version of the manuscript for publication. All authors are accountable for all aspects of the work in ensuring that questions related to the accuracy or integrity of any part of the work are appropriately investigated and resolved.

## Conflict of Interest Statement

The authors declare that the research was conducted in the absence of any commercial or financial relationships that could be construed as a potential conflict of interest.

## Footnotes

^1^Two nouns of the original stimulus material of Bayer et al. (2012a) had to be replaced because of their ambiguous phonology.

^2^http://www.biosemi.com/faq/cms&drl.htm
